# Dimethyl 4,4′-(pyridine-2,6-diyl)dibenzoate

**DOI:** 10.1107/S160053681004362X

**Published:** 2010-10-31

**Authors:** Yue Cui, Qian Gao, Huan-Huan Wang, Lin Wang, Ya-Bo Xie

**Affiliations:** a College of Environmental and Energy Engineering, Beijing University of Technology, Beijing 100124, People’s Republic of China

## Abstract

The title mol­ecule, C_21_H_17_NO_4_, reveals axial symmetry, with the pyridine N atom located on a crystallographic twofold axis. The mol­ecule is dish-shaped, with dihedral angles between the benzene and pyridine rings of 24.643 (1) and 24.797 (1)°, respectively. The –COO plane and the benzene ring are almost coplanar [dihedral angle = 5.286 (1)°].

## Related literature

For applications of the title compound, see: Boyle *et al.* (2010 ▶). For the synthesis, see: Li & Zhou (2009 ▶).
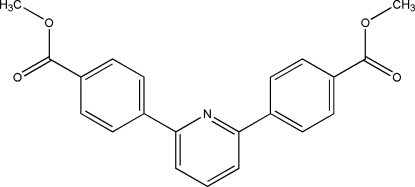


## Experimental

### 

#### Crystal data

C_21_H_17_NO_4_
*M* *_r_* = 347.36
Orthorhombic, 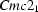

*a* = 34.296 (10) Å
*b* = 7.401 (2) Å
*c* = 6.623 (2) Å
*V* = 1681.1 (9) Å^3^
*Z* = 4
Mo *K*α radiation
μ = 0.10 mm^−1^
*T* = 296 K
0.60 × 0.40 × 0.36 mm

#### Data collection

Bruker APEXII CCD diffractometer
Absorption correction: multi-scan (*SADABS*; Bruker, 1998 ▶) *T*_min_ = 0.945, *T*_max_ = 0.966
7265 measured reflections
2264 independent reflections
2151 reflections with *I* > 2σ(*I*)
*R*_int_ = 0.035

#### Refinement

*R*[*F* ^2^ > 2σ(*F* ^2^)] = 0.037
*wR*(*F* ^2^) = 0.109
*S* = 1.05
2264 reflections
122 parameters
1 restraint
H-atom parameters constrained
Δρ_max_ = 0.25 e Å^−3^
Δρ_min_ = −0.16 e Å^−3^

### 

Data collection: *SMART* (Bruker, 1998 ▶); cell refinement: *SAINT* (Bruker, 1998 ▶); data reduction: *SAINT*; program(s) used to solve structure: *SHELXS97* (Sheldrick, 2008 ▶); program(s) used to refine structure: *SHELXL97* (Sheldrick, 2008 ▶); molecular graphics: *SHELXTL* (Sheldrick, 2008 ▶); software used to prepare material for publication: *SHELXTL*.

## Supplementary Material

Crystal structure: contains datablocks I, global. DOI: 10.1107/S160053681004362X/kp2283sup1.cif

Structure factors: contains datablocks I. DOI: 10.1107/S160053681004362X/kp2283Isup2.hkl

Additional supplementary materials:  crystallographic information; 3D view; checkCIF report

## Figures and Tables

**Fig. 1.**
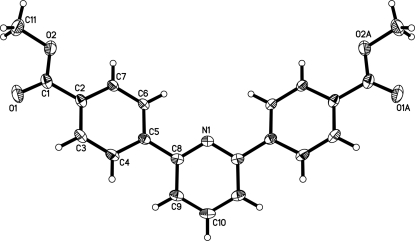
The molecular structure of the title compound with displacement ellipsoids drawn at the 30% probability level, H atoms are shown as small circles of arbitrary radius. Symmetry code: A = -x, y, z.

## supplementary crystallographic information

### Comment

Pyridine-type compounds and their derivatives have been extensively investigated because of their wide application for the synthesis of various complex compounds (Boyle *et al.*, 2010). Herein, we report the crystal structure of the title compound (Fig. 1), dimethyl 4,4'-pyridine-2,6-diyldibenzoate.

The title compound, C_21_H_17_NO_4_, was synthesised by the reaction of 2,6-dibromopyridine and 4-methoxycarbonylphenylboronic acid. The molecule reveals a crystallographic twofold axis with the N atom lying on a special position - the rotation twofold axis. The dihedral angles between the benzene ring and the pyridine ring are 24.643 (1)° and 24.797 (1)°, respectively. The –COO plane and the benzene ring are almost coplanar, and the dihedral angles are 5.363 (1)° and 4.794 (1)°, respectively.

### Experimental

The title compound was synthesised according to the reported procedure (Li & Zhou, 2009). Colourless single crystals suitable for X-ray diffraction were obtained by recrystallisation from a solvents mixture of ethyl acetate and hexane.

### Refinement

All H atoms were placed in calculated positions with C—H = 0.93–0.96 Å, and refined as riding with *U*_iso(H)_ = 1.2*U*_eq_(C) or 1.5*U*_eq_(C_methyl_).

### Crystal data

**Table:**

C_21_H_17_NO_4_	*F*(000) = 728
*M**_r_* = 347.36	*D*_x_ = 1.372 Mg m^−^^3^
Orthorhombic, *C**m**c*2_1_	Mo *K*α radiation, λ = 0.71073 Å
Hall symbol: C 2c -2	Cell parameters from 4869 reflections
*a* = 34.296 (10) Å	θ = 2.4–30.5°
*b* = 7.401 (2) Å	µ = 0.10 mm^−^^1^
*c* = 6.623 (2) Å	*T* = 296 K
*V* = 1681.1 (9) Å^3^	Block, colourless
*Z* = 4	0.60 × 0.40 × 0.36 mm

### Data collection

**Table:**

Bruker APEXII CCD diffractometer	2264 independent reflections
Radiation source: fine-focus sealed tube	2151 reflections with *I* > 2σ(*I*)
graphite	*R*_int_ = 0.035
φ and ω scans	θ_max_ = 30.5°, θ_min_ = 2.4°
Absorption correction: multi-scan (*SADABS*; Bruker, 1998)	*h* = −48→44
*T*_min_ = 0.945, *T*_max_ = 0.966	*k* = −10→10
7265 measured reflections	*l* = −9→7

### Refinement

**Table:**

Refinement on *F*^2^	Secondary atom site location: difference Fourier map
Least-squares matrix: full	Hydrogen site location: inferred from neighbouring sites
*R*[*F*^2^ > 2σ(*F*^2^)] = 0.037	H-atom parameters constrained
*wR*(*F*^2^) = 0.109	*w* = 1/[σ^2^(*F*_o_^2^) + (0.069*P*)^2^ + 0.231*P*] where *P* = (*F*_o_^2^ + 2*F*_c_^2^)/3
*S* = 1.05	(Δ/σ)_max_ < 0.001
2264 reflections	Δρ_max_ = 0.25 e Å^−^^3^
122 parameters	Δρ_min_ = −0.16 e Å^−^^3^
1 restraint	Absolute structure: Flack (1983), with how many Friedel pairs?
Primary atom site location: structure-invariant direct methods	Flack parameter: 1.2 (12)

### Special details

**Table:**

Geometry. All e.s.d.'s (except the e.s.d. in the dihedral angle between two l.s. planes) are estimated using the full covariance matrix. The cell e.s.d.'s are taken into account individually in the estimation of e.s.d.'s in distances, angles and torsion angles; correlations between e.s.d.'s in cell parameters are only used when they are defined by crystal symmetry. An approximate (isotropic) treatment of cell e.s.d.'s is used for estimating e.s.d.'s involving l.s. planes.
Refinement. Refinement of *F*^2^ against ALL reflections. The weighted *R*-factor *wR* and goodness of fit *S* are based on *F*^2^, conventional *R*-factors *R* are based on *F*, with *F* set to zero for negative *F*^2^. The threshold expression of *F*^2^ > σ(*F*^2^) is used only for calculating *R*-factors(gt) *etc*. and is not relevant to the choice of reflections for refinement. *R*-factors based on *F*^2^ are statistically about twice as large as those based on *F*, and *R*-factors based on ALL data will be even larger.

### Fractional atomic coordinates and isotropic or equivalent isotropic displacement parameters (Å^2^)

**Table:**

	*x*	*y*	*z*	*U*_iso_*/*U*_eq_	
O1	0.20583 (3)	0.17226 (19)	0.8336 (2)	0.0693 (4)	
N1	0.0000	0.24751 (18)	0.5426 (2)	0.0316 (3)	
C1	0.17708 (3)	0.24851 (18)	0.8930 (2)	0.0429 (3)	
O2	0.17487 (3)	0.32976 (15)	1.0717 (2)	0.0535 (3)	
C2	0.13992 (3)	0.25470 (16)	0.77804 (19)	0.0360 (3)	
C3	0.13855 (4)	0.16771 (18)	0.5923 (2)	0.0424 (3)	
H3	0.1609	0.1139	0.5408	0.051*	
C4	0.10431 (4)	0.16036 (18)	0.4834 (2)	0.0416 (3)	
H4	0.1038	0.1010	0.3597	0.050*	
C5	0.07037 (3)	0.24139 (14)	0.55745 (17)	0.0323 (2)	
C6	0.07197 (3)	0.33053 (14)	0.74267 (19)	0.0328 (2)	
H6	0.0496	0.3854	0.7936	0.039*	
C7	0.10641 (3)	0.33846 (14)	0.8520 (2)	0.0343 (2)	
H7	0.1072	0.3996	0.9746	0.041*	
C8	0.03362 (3)	0.23143 (15)	0.44012 (18)	0.0330 (3)	
C9	0.03455 (4)	0.20276 (19)	0.2306 (2)	0.0405 (3)	
H9	0.0583	0.1927	0.1634	0.049*	
C10	0.0000	0.1898 (3)	0.1258 (3)	0.0439 (4)	
H10	0.0000	0.1725	−0.0133	0.053*	
C11	0.21015 (5)	0.3231 (3)	1.1922 (3)	0.0644 (5)	
H11A	0.2192	0.2006	1.2007	0.097*	
H11B	0.2048	0.3677	1.3254	0.097*	
H11C	0.2299	0.3967	1.1303	0.097*	

### Atomic displacement parameters (Å^2^)

**Table:**

	*U*^11^	*U*^22^	*U*^33^	*U*^12^	*U*^13^	*U*^23^
O1	0.0334 (4)	0.0929 (10)	0.0817 (9)	0.0119 (5)	0.0066 (5)	−0.0199 (8)
N1	0.0386 (6)	0.0284 (6)	0.0279 (7)	0.000	0.000	−0.0001 (5)
C1	0.0309 (5)	0.0434 (7)	0.0545 (9)	−0.0021 (4)	0.0080 (5)	−0.0022 (5)
O2	0.0386 (4)	0.0624 (7)	0.0597 (7)	0.0080 (4)	−0.0070 (5)	−0.0122 (5)
C2	0.0305 (4)	0.0341 (6)	0.0435 (7)	−0.0025 (4)	0.0093 (4)	−0.0004 (4)
C3	0.0374 (5)	0.0446 (7)	0.0453 (8)	0.0031 (5)	0.0150 (5)	−0.0053 (5)
C4	0.0450 (6)	0.0423 (6)	0.0374 (7)	0.0019 (5)	0.0111 (5)	−0.0080 (5)
C5	0.0371 (5)	0.0294 (5)	0.0305 (6)	−0.0024 (4)	0.0065 (5)	0.0006 (4)
C6	0.0316 (5)	0.0340 (5)	0.0328 (6)	0.0008 (4)	0.0076 (4)	−0.0022 (4)
C7	0.0329 (5)	0.0356 (5)	0.0344 (6)	−0.0004 (4)	0.0060 (5)	−0.0053 (4)
C8	0.0419 (6)	0.0275 (5)	0.0298 (6)	−0.0016 (4)	0.0034 (4)	0.0001 (4)
C9	0.0513 (7)	0.0404 (6)	0.0297 (6)	−0.0019 (5)	0.0072 (5)	−0.0001 (5)
C10	0.0669 (12)	0.0405 (9)	0.0244 (8)	0.000	0.000	−0.0014 (7)
C11	0.0470 (7)	0.0742 (11)	0.0722 (13)	0.0074 (8)	−0.0192 (8)	−0.0093 (9)

### Geometric parameters (Å, °)

**Table:**

O1—C1	1.2025 (16)	C5—C8	1.4823 (15)
N1—C8^i^	1.3433 (13)	C6—C7	1.3866 (17)
N1—C8	1.3433 (13)	C6—H6	0.9300
C1—O2	1.3294 (19)	C7—H7	0.9300
C1—C2	1.4854 (17)	C8—C9	1.4041 (18)
O2—C11	1.4503 (18)	C9—C10	1.3768 (17)
C2—C3	1.3895 (19)	C9—H9	0.9300
C2—C7	1.3945 (14)	C10—C9^i^	1.3768 (17)
C3—C4	1.379 (2)	C10—H10	0.9300
C3—H3	0.9300	C11—H11A	0.9600
C4—C5	1.3981 (16)	C11—H11B	0.9600
C4—H4	0.9300	C11—H11C	0.9600
C5—C6	1.3940 (17)		
			
C8^i^—N1—C8	118.29 (15)	C5—C6—H6	119.6
O1—C1—O2	123.39 (14)	C6—C7—C2	119.98 (11)
O1—C1—C2	123.39 (14)	C6—C7—H7	120.0
O2—C1—C2	113.18 (10)	C2—C7—H7	120.0
C1—O2—C11	115.28 (12)	N1—C8—C9	122.14 (12)
C3—C2—C7	119.28 (11)	N1—C8—C5	117.42 (11)
C3—C2—C1	117.95 (11)	C9—C8—C5	120.42 (11)
C7—C2—C1	122.73 (12)	C10—C9—C8	119.31 (13)
C4—C3—C2	120.65 (11)	C10—C9—H9	120.3
C4—C3—H3	119.7	C8—C9—H9	120.3
C2—C3—H3	119.7	C9^i^—C10—C9	118.78 (17)
C3—C4—C5	120.59 (12)	C9^i^—C10—H10	120.6
C3—C4—H4	119.7	C9—C10—H10	120.6
C5—C4—H4	119.7	O2—C11—H11A	109.5
C6—C5—C4	118.59 (11)	O2—C11—H11B	109.5
C6—C5—C8	121.23 (9)	H11A—C11—H11B	109.5
C4—C5—C8	120.18 (10)	O2—C11—H11C	109.5
C7—C6—C5	120.88 (10)	H11A—C11—H11C	109.5
C7—C6—H6	119.6	H11B—C11—H11C	109.5
			
O1—C1—O2—C11	0.9 (2)	C5—C6—C7—C2	−0.78 (16)
C2—C1—O2—C11	178.41 (13)	C3—C2—C7—C6	1.58 (17)
O1—C1—C2—C3	0.0 (2)	C1—C2—C7—C6	−176.21 (11)
O2—C1—C2—C3	−177.60 (12)	C8^i^—N1—C8—C9	1.5 (2)
O1—C1—C2—C7	177.77 (15)	C8^i^—N1—C8—C5	−177.32 (8)
O2—C1—C2—C7	0.22 (18)	C6—C5—C8—N1	−24.37 (16)
C7—C2—C3—C4	−1.41 (18)	C4—C5—C8—N1	155.51 (12)
C1—C2—C3—C4	176.48 (12)	C6—C5—C8—C9	156.81 (12)
C2—C3—C4—C5	0.4 (2)	C4—C5—C8—C9	−23.31 (16)
C3—C4—C5—C6	0.39 (18)	N1—C8—C9—C10	−0.29 (19)
C3—C4—C5—C8	−179.49 (11)	C5—C8—C9—C10	178.47 (14)
C4—C5—C6—C7	−0.21 (16)	C8—C9—C10—C9^i^	−0.9 (3)
C8—C5—C6—C7	179.67 (10)		

Symmetry codes: (i) −*x*, *y*, *z*.
